# Spin Transition Sensors Based on β-Amino-Acid 1,2,4-Triazole Derivative

**DOI:** 10.3390/ijms12085339

**Published:** 2011-08-18

**Authors:** Marinela M. Dîrtu, France Schmit, Anil D. Naik, Aurelian Rotaru, J. Marchand-Brynaert, Yann Garcia

**Affiliations:** 1 Institute of Condensed Matter and Nanosciences, Université Catholique de Louvain, Place L. Pasteur 1, 1348 Louvain-la-Neuve, Belgium; E-Mails: marinela.dirtu@uclouvain.be (M.M.D.); france.schmit@gmail.com (F.S.); anil.naik@uclouvain.be (A.D.N.); jacqueline.marchand@uclouvain.be (J.M.-B.); 2 Department of Electrical Engineering and Computer Science, “Stefan cel Mare” University, University Street 13, Suceava 720229, Romania; E-Mail: aurelian.rotaru@gmail.com

**Keywords:** spin crossover, aminoacid triazoles, iron(II), coordination polymers

## Abstract

A β-aminoacid ester was successfully derivatized to yield to 4H-1,2-4-triazol-4-yl-propionate (**βAlatrz**) which served as a neutral bidentate ligand in the 1D coordination polymer [Fe(**βAlatrz**)_3_](CF_3_SO_3_)_2_·0.5H_2_O (**1**·**0.5H_2_O**). The temperature dependence of the high-spin molar fraction derived from ^57^Fe Mossbauer spectroscopy recorded on cooling below room temperature reveals an exceptionally abrupt single step transition between high-spin and low-spin states with a hysteresis loop of width 4 K (*T*_c_^↑^ = 232 K and *T*_c_^↓^ = 228 K) in agreement with magnetic susceptibility measurements. The material presents striking reversible thermochromism from white, at room temperature, to pink on quench cooling to liquid nitrogen, and acts as an alert towards temperature variations. The phase transition is of first order, as determined by differential scanning calorimetry, with transition temperatures matching the ones determined by SQUID and Mössbauer spectroscopy. The freshly prepared sample of **1**·**0.5H_2_O**, dried in air, was subjected to annealing at 390 K, and the obtained white compound [Fe(**βAlatrz**)_3_](CF_3_SO_3_)_2_ (**1**) was found to exhibit a similar spin transition curve however much temperature was increased by (*T*_c_^↑^ = 252 K and *T*_c_^↓^ = 248 K). The removal of lattice water molecules from **1**·**0.5H_2_O** is not accompanied by a change of the morphology and of the space group, and the chain character is preserved. However, an internal pressure effect stabilizing the low-spin state is evidenced.

## 1. Introduction

Bistable molecular systems, particularly materials exhibiting captivating scenario of spin crossover (SCO) [1], are versatile switchable units in the thriving field of molecular electronics [2]. In a typical SCO material, the electron repositioning via singlet-quintet transitions is substantiated to be technologically significant [2]. Indeed, the reversible electron transfer from a diamagnetic low-spin (LS, ^1^A_1g_) state to a thermally populated paramagnetic high-spin (HS, ^5^T_2g_) state is recognized as an entropy driven process and could be addressed thermally, optically, electrically and under pressure/shock with highly profound spectroscopic, optical, magnetic, dielectric readout signal [3]. In the solid state, the presence of intra and intermolecular interactions acts as communication media between iron centers promoting cooperative first order spin transitions leading to a large memory domain [4] that can be suitable for potential applications [5]. Indeed, a SCO compound meeting display and data processing requirements would, in addition, have a good shelf life and an easily detectable optical response, and would ideally operate near room temperature [6,7].

**Chart 1.**
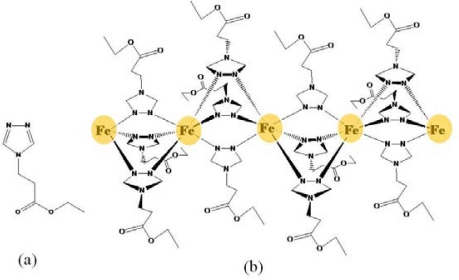
Molecular structure of (**a**) ethyl 4H-1,2,4-triazol-4-yl-propionate (**βAlatrz**); and (**b**) complex [Fe(**βAlatrz**)_3_]^2+^.

Applications envisioned in these fields largely depend on molecular conformations precursors adopt during the coordination process which directs structure-properties relationships. In some cases, magnetic properties can even be modified by a structural perturbation as demonstrated on a 1D polyelectrolyte system [8–11]. Our interest in amino acid derivatization was fuelled by promising results shown by 1,2,4-triazole-carboxylate derivatives in synthetic chemistry [12], spin crossover area [13], nanoporous MOFs [14], biological interest in several metallo-proteases [15] and ‘soft’ sacrificial precursors to produce CdO with shape and phase selectivity [16]. As a continuation of our work on amino acid functionalization, [12] ethyl-4H-1,2,4-triazol-4-yl-acetate was used as a prospective precursor for magnetic sensors [13]. Here we introduce a new tailored 4-R-1,2,4-triazole from β-amino-acid, namely 4H-1,2,4-triazol-4-yl-propionate (**βAlatrz**) (Chart 1). The reason for introducing β-Alanine ethyl ester substituted on the 4 position of a 1,2,4-triazole core was to use an appropriate length of substituent which compromises the distance between 1D chains in complexes which can introduce supramolecular interactions with H-acceptors substituent. Thus, the spin transition (ST) properties of two one-dimensional Fe^II^ chains, [Fe(**βAlatrz**)_3_](CF_3_SO_3_)_2_·0.5H_2_O (**1**·**0.5H_2_O**) and its dehydrated form, [Fe(**Alatrz**)_3_](CF_3_SO_3_)_2_ (**1**) were studied.

## 2. Results and Discussion

### 2.1. Preparation and Characterization of **1**·**0.5H_2_O** and **1**

The transamination reaction used in the synthesis of ethyl-4H-1,2,4-triazol-4-yl-acetate and 4H-1,2,4-triazol-4-yl acetic acid [12] proved to be a good synthetic strategy to build a β-amino-acid triazole. The synthetic route, starting with β-Alanine ethyl ester hydrochloride and *N*,*N*-Dimethylformamide-azine, helped us to obtain a new molecule, **βAlatrz**, with a good yield. The synthesis time is relatively longer than the synthesis of previous ligands and the final product had to be purified by “flash” column chromatography.

**Figure 1.**
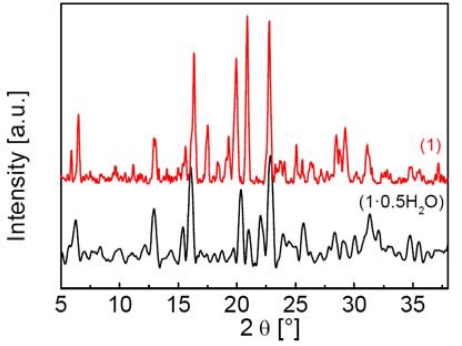
Top: X-ray powder diffraction pattern of **1**, (thermally treated compound in TGA); bottom: diffractogram of **1**·**0.5H_2_O** (freshly prepared).

A 1D coordination polymer was obtained as a white powder by reaction of the corresponding Fe^II^ inorganic precursor, prepared in air, [Fe(H_2_O)_6_](CF_3_SO_3_)_2_ [17] with a methanolic solution of **βAlatrz**. This complex was successfully characterized by elemental analysis, TGA-DTA analyses, atomic absorption (AAS), X-Ray powder diffraction, IR, Raman, SEM, DSC, SQUID magnetometry and ^57^Fe Mössbauer spectroscopy. The thermogravimetric and elemental analyses reveal the presence of guest water molecules, affording the following general formula [Fe(**βAlatrz**)_3_](CF_3_SO_3_)_2_·0.5H_2_O (**1**·**0.5H_2_O**). Compound **1**·**0.5H_2_O** was dehydrated by annealing, leading to a new compound, [Fe(**βAlatrz**)_3_](CF_3_SO_3_)_2_ (**1**), which was also characterized to reveal the influence of solvent molecules on the spin state. **1**·**0.5H_2_O** presents a rather crystalline character as revealed from X-ray powder diffraction (XRPD) patterns (Figure 1).

**Figure 2.**
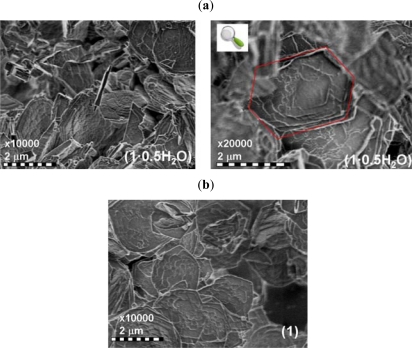
(**a**) SEM images of microcrystalline particles of **1**·**0.5H_2_O** at 293 K; a selected crystal is highlighted in red; and (**b**) SEM analysis of **1** confirms the framework integrity preservation after thermal treatment.

**Figure 3.**
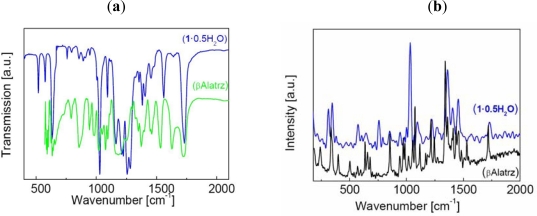
(**a**) IR and (**b**) Raman spectra of the ligand (**βAlatrz**) and complex (**1**·**0.5H_2_O**), over the range 400–2,100 cm^−1^ and 180–2,000 cm^−1^, respectively. The IR and Raman spectra for **1**·**0.5H_2_O** and **1** (not shown) are identical, except around 3,500 cm^−1^ with ν_(O–H)_ of water molecules clearly identified for **1**·**0.5H_2_O**.

This result is also illustrated by the SEM analysis with blocks of hexagonal shape of about 2 μm widths (Figure 2). FT-IR and Raman analyses were performed: (i) to confirm the identity of the ligand framework before and upon complexation as the ester functionality is susceptible to hydrolysis; (ii) to ascertain the coordination mode of iron to the ligand and the presence of the trifluoromethane sulfonate in the crystal lattice (Figure 3).

Two sharp IR bands at 3,118(s) and 2,983(m) cm^−1^ of **βAlatrz**, are assigned to the νCH_2_ modes [18]. The carboxylic group of ester shows two stretching vibrations corresponding to (C=O) and (C–O) at 1,728(s) and 1,205(s) cm^−1^, respectively, which are almost unchanged (1,732 and 1,212 cm^−1^) in **1**·**0.5H_2_O** ruling out the possible ester hydrolysis and also confirming the non-involvement of carboxylic ester group in coordination. For **βAlatrz**, the band assigned to ring torsion of triazole at ν = 634(m) cm^−1^, the ν_C=N_ stretching vibration at 1,535(m) cm^−1^ and the N–N stretching band at ν = 1,022(s) cm^−1^, are all shifted upon complexation in **1**·**0.5H_2_O** to 632 cm^−1^, ν_C=N_ = 1,560 cm^−1^, and ν_N-N_ = 1,028 cm^−1^, respectively.

These values confirm the coordination of the iron to the 1,2,4-triazole ring [19,20]. Indeed, the characteristic bands of the ligand are not only present in the spectra of the complexes but are also shifted towards larger wave numbers, from 3 to 15 cm^−1^, respectively. This energy increase is due to the deformation of the ligand upon coordination of the metal, the ligand molecule being indeed more constrained to perform vibration and twisting movements [19,21].

For a better understanding of the molecular structure of these ligands, Raman spectra were also collected (Figure 3). The band present at 1,728 cm^−1^ for **βAlatrz** is assigned to a C=O vibration. In complexes this band is retained and appears around 1,732 cm^−1^. The C–C stretching is also active in Raman as a medium band at 1,098 cm^−1^ for **βAlatrz** and appears in the same wavenumber in **1**·**0.5H_2_O**. Presence of the monovalent counter-anion is also confirmed by IR and Raman: *IR* (cm^−1^) υ(S–O)∼1,283(s), (**1**·**0.5H_2_O**); *Raman* (cm^−1^) υ_(S–O)_∼1,034(s), 760(m) (**1**·**0.5H_2_O**) [22]. ^57^Fe Mössbauer spectroscopy confirm for **1**·**0.5H_2_O** the presence of one Fe^II^N_6_ site, and exclude any oxidation product of iron. These spectroscopic data support a linear chain structure with Fe^II^ ions linked by triple *N*1,*N*2-1,2,4-triazole bridges [23,24].

**Figure 4.**
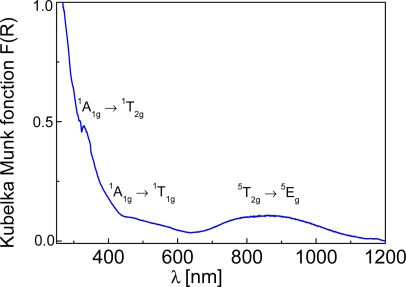
UV-Vis diffuse reflectance spectrum of **1**·**0.5H_2_O** showing d–d transitions at 293 K.

**1·0.5H_2_O** was prepared as a white powders and presents a reversible thermochromism on cooling to pink (Figure 4). These colors depend on the spin state of the Fe^II^ centers, as the ST involves a change in the electronic configuration which modifies the absorption spectrum of the complex.

The white color of **1**·**0.5H_2_O**, is due to the location of the spin-allowed lowest energy d-d transition, ^5^T_2g_ → ^5^E_g_, for the HS sites in the near infrared region (∼11,500 cm^−1^) [25,26]. Two supplementary bands, corresponding to the ^1^A_1g_ → ^1^T_1g_ and ^1^A_1g_ → ^1^T_2g_ d-d transitions of LS Fe^II^ sites are observed at ∼18,727 cm^−1^ and ∼30,300 cm^−1^, respectively suggesting that some proportion of Fe^II^ may be in the LS state at room temperature. The ligand field strengths for the HS and the LS state, given by equations 10D_q_^HS^ = E(^5^E) − E(^5^T_2_) and 10D_q_^LS^ = E(^1^T) − E(^1^A_1_) + (E(^1^T_2_) − E(^1^T_1_))/4, [25,26] allowed us to estimate 10D_q_^HS^ and 10D_q_^LS^ to be approximately 11,500 cm^−1^ and 26,302 cm^−1^, respectively. These values are characteristic for SCO complexes [25,26]. Presence of LS state at room temperature has been confirmed by ^57^Fe Mössbauer spectroscopy (vide infra).

The reversibility of the rehydration/dehydration process within a given framework structure with the change of crystallinity can play an important role on the SCO properties [27]. This process was investigated for **1**·**0.5H_2_O** by thermogravimetric analysis, and the XRD powder pattern and SEM imaging recorded after annealing treatment. The sample was first deposited in a crucible and slowly heated at 1 K/min in air atmosphere (air flow 130 mL/min) to the temperature of complete dehydration (390 K) leading to **1**. After a short standby period, the sample was cooled (1 K/min) slowly to reach room temperature revealing no rehydration. Thus the dehydration-rehydration process is irreversible and **1** is air stable which will ease any further physical studies on this compound. Comparison of the powder XRD patterns (Figure 1) and SEM images (Figure 2) of **1**·**0.5H_2_O** and **1** allow to exclude any framework rupture. Indeed, these two compounds are isostructural, **1** showing higher peak intensities indicating a better crystalline character. No deterioration is observed by SEM.

### 2.2. SQUID Magnetometry

**Figure 5.**
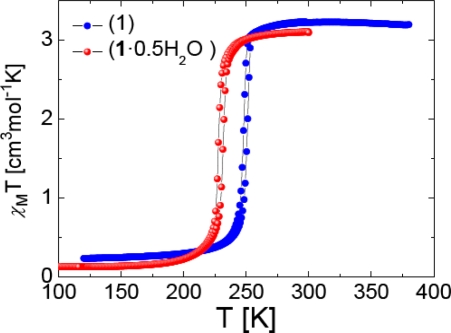
Thermal variation of *χ*_M_*T* of **1**·**0.5H_2_O** (depicted in red) and **1** (depicted in blue).

The temperature dependent magnetic properties of **1**·**0.5H_2_O** and **1** were determined by temperature-dependent susceptibility measurements using a SQUID magnetometer operating at 1000 Oe (Figure 5).

For complex **1**·**0.5H_2_O**, χ_M_*T* is 3.10 cm^3^ K mol^−1^ at 300 K, which is in the region expected for an Fe^II^ complex essentially in the HS state, with a small % of LS species. Upon cooling, *χ*_M_*T* remains almost constant until 240 K where it drops sharply to *T_c_*^↓^ = 228 K down to ∼0.14 cm^3^ K mol^−1^ at 77 K, which is typical for a diamagnetic Fe^II^ complex with possibly a few HS ions.

Upon warming, the χ_M_*T* curve differs slightly revealing a small hysteresis width (4 K) at *T_c_*^↑^ = 232 K for this very sharp spin transition. An hysteretic behavior of width 4 K was observed too for **1** with a ST shifted upwards (*T_c_*^↓^ = 248 K and *T_c_*^↑^ = 252 K). The same curve shape is observed too which confirms the preservation of the 1D chain character [27] and does not support the appearance of a structural phase transition following the annealing process. The stabilization of the LS state upon water release is uncommon for this family of complexes [28] and was only observed for the 1D chain compounds [Fe(4-(2′-hydroxyethyl)-1,2,4-triazole)_3_](anion)_2_·nH_2_O (anion = ClO_4_^−^, n = 2, 0; anion = I^−^, n = 1, 0) [29], [Fe(4-amino-1,2,4-triazole)_3_](anion)·nH_2_O (anion = TiF_6_^2−^, n = 1, 0.5; anion = ZrF_6_^2−^, n = 0.5, 0) [23] as well as for [Fe(ethyl-4H-1,2,4-triazol-4-yl-acetate)_3_](ClO_4_)_2_·nCH_3_OH (n = 1,0) but with methanol as solvent [13]. The effect of the release of non-coordinated solvent molecules on the spin state can be translated here as an internal positive pressure effect [5,23].

### 2.3. ^57^Fe Mössbauer Spectroscopy

**Figure 6.**
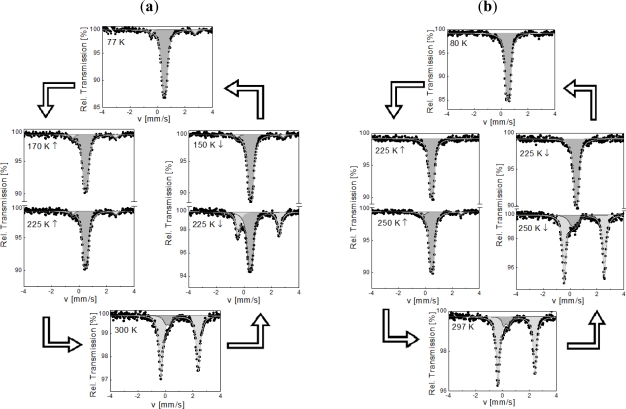
^57^Fe Mössbauer spectra cycles for **1**·**0.5H_2_O** (**a**) and **1** (**b**) at selected temperatures. Grey and dark grey correspond to the high-spin (HS) and low-spin (LS) doublets, respectively.

An inspection of electronic and structural features for **1**·**0.5H_2_O** and **1** using ^57^Fe Mössbauer spectroscopy was undertaken over the temperature range 77–300 K (Figure 6). At 77 K, the spectrum of **1**·**0.5H_2_O** consists of two quadrupole doublets of different resonance area fractions. The major quadrupole doublet with isomer shift *δ*^LS^ = 0.51(2) mm/s and quadrupole splitting, *ΔE_Q_^LS^* = 0.23(2) mm/s correspond to the LS state of Fe^II^. The presence of an LS quadrupole splitting stems from a lattice contribution to the electric field gradient and therefore reveals a distorted character for the LS octahedron as expected within a chain, where constraints may not be negligible [30]. Another doublet, corresponding to HS Fe^II^ ions of weaker population (7%), with parameters (*δ^HS^* = 1.14(1) mm/s and *ΔE_Q_^HS^* = 3.15(2) mm/s) confirms the incomplete nature of the ST at 77 K. Upon warming to 300 K, the intensity of the HS doublet slowly increases to 8% at 225 K, after which it increases dramatically to 87.5% at 300 K, confirming an incomplete thermally induced LS → HS conversion for a single Fe^II^ site. The presence of LS ions at room temperature could be related to crystal defects or end of chains. The hysteresis effect is clearly evidenced at 225 K (Figure 6a).

At 80 K, the spectrum of **1** shows a single LS quadrupole doublet (*δ*^LS^ = 0.52(1) mm/s and *ΔE_Q_*^LS^ = 0.25(2) mm/s) indicating a complete spin transition. The compound remains mostly in the LS state on warming up to 250 K, after which a second quadrupole doublet attributed to HS Fe^II^ ions grows in intensity (e.g., at 297 K, *δ*^HS^ = 1.03(1) mm/s and *ΔE_Q_*^HS^ = 2.75(1) mm/s). The asymmetry of the lines observed in the HS state is attributed to a texture effect. Upon cooling to low temperature, the reverse situation is observed with a clear hysteresis effect at 250 K (Figure 6b).

The isomer shift (*δ*^LS^∼0.51(1) mm/s at 77–80 K and *ΔE_Q_*^HS^ = 1.03(1) mm/s at 297–300 K) is not affected by the dehydration process which indicates that non coordinated water molecules are not H-bonded to the triazole ligand [31], but should be located at a remote position to the complex in the crystal lattice, either isolated or hydrogen bonded to the sulfonate group of the non coordinated anions. Their releases have no influence on the structural organization as demonstrated by the similarity in X-ray powder diffraction data. However, a clear influence on the transition temperatures, *i.e.*, on the respective energy levels of the HS and LS states [25], has been detected.

### 2.4. Differential Scanning Calorimetry

**Figure 7.**
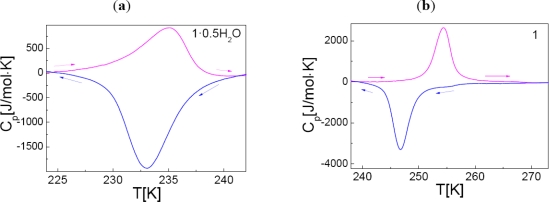
Heat capacity profiles for **1**·**0.5H_2_O** and **1**. The arrows indicate the cooling (←) and warming (→) modes, respectively.

Compound **1**·**0.5H_2_O** and **1** were investigated by differential scanning calorimetry over the temperature range 100–300 K, at 10 K/min for both cooling and heating modes (Figure 7).

An endothermic peak is observed on warming for **1**·**0.5H_2_O** at *T_max_*^↑^ = 235(1) K and an exothermic peak is recorded at *T_max_*^↓^ = 233(1) K, on cooling. These peaks correspond to a first-order phase transition in agreement with the transition temperatures determined by both SQUID and Mössbauer spectroscopy. The enthalpy and entropy variations associated to the active SCO centers are *ΔH* = 12(2) kJ/mol and *ΔS* = 51(2) J/mol/K. A similar profile, although with more abrupt peaks, was detected for **1** with phase transitions shifted upwards to *T_max_*^↑^ = 254(1) K and *T_max_*^↓^ = 247(1) K. The peaks are separated by a narrow temperature domain, which is indicative of the presence of a hysteresis loop. The enthalpy and entropy associated to the ST, considering only the switching sites, were derived as follows: *ΔH* = 13(1) kJ/mol and *ΔS* = 59(2) J/mol/K.

## 3. Experimental Section

### 3.1. Chemicals

All reagents and solvents were used as received from commercial source: benzene (Fluka analytical), Methanol (VWR), SOCl_2_ (Sigma-Aldrich), glycine ethyl ester hydrochloride (ACROS), CF_3_SO_3_H (ACROS), Fe powder (Merck). Ethyl 4H-1,2,4-triazol-4-yl-propionate (**βAlatrz**) were prepared by a similar method described in reference [12].

#### 3.2.1. Ethyl 4H-1,2,4-Triazol-4-yl-Propionate (βAlatrz)

*N*,*N*-Dimethylformamide azine dihydrochloride (**I**) and its free base (**II**) were obtained following the reported method [12]. The free base was recrystallized twice from benzene with charcoal and used in transamination reactions [12]. To a suspension of *β-alanine ethyl ester hydrochloride* (3 g, 19.53 mmol) in benzene (100 mL) at approx. 60 °C was added solid **II** (2.14 g, 15.04 mmol) with stirring, obtaining a transparent yellow solution. The mixture was refluxed then (at 130 °C) for 93 h with vigorous stirring. The reaction was monitored by NMR analysis at interval of time. Finally, the solvent was removed under vacuum and a chromatographic purification (SiO_2_, CH_2_Cl_2_ → 5% isopropanol in CH_2_Cl_2_) of the yellow oil gave pale yellow oil. Yield 2 g (60%). ^1^H NMR (300 MHz, CDCl_3_, 298 K): δ = 8.25 (s, 2H), 4.34 (*t*, 3H, *J* = 6.17 Hz), 4.14 (*dd*, 2H, *J* = 7.10 Hz & 7.15 Hz), 2.78 (*t*, 2H, *J* = 6.17 Hz), 1.23 (*t*, 3H, *J* = 7.16 Hz). ^13^C NMR (300 MHz, CDCl_3_ 298 K): δ = 170.1, 143, 61.6, 40.6, 35.5, 14.1. MS: *m*/*z* = 170.03 (M + H^+^). FTIR (thin film from CH_2_Cl_2_ on ZnSe HATR through plate, cm^−1^): 3,118(s), 1,728(vs), 1,535(s), 1,236(s), 1,205(s), 1,186(s), 1,022(m), 637(s). Anal. Calcd. for C_7_H_11_N_3_O_2_ (169.18 g/mol): C, 49.70, H, 6.55, N, 24.84; Found C, 49.7, H, 6.81, N, 22.61.

#### 3.2.2. [Fe(βAlatrz)_3_](CF_3_SO_3_)_2_·0.5H_2_O (**1***·***0.5H_2_O**)

[Fe(H_2_O)_6_](CF_3_SO_3_)_2_ was first synthesized as a very pale green powder using a described procedure [16], starting with an aqueous solution (1 mL) containing an iron powder in excess (2 g) carefully mixed to triflic acid (5 mL, 56.5 mmol). Yield: 9.8 g, 74%. [Fe(H_2_O)_6_](CF_3_SO_3_)_2_ (183.2 mg, 0.396 mmol) was dissolved in CH_3_OH (5 mL) with a pinch of ascorbic acid and added to the above solution of **βAlatrz** (205.7 mg, 1.216 mmol) dissolved in CH_3_OH (5 mL). The mixture was stirred for 15 min at room temperature, after which a white precipitate was obtained. It was filtered, washed with CH_3_OH (2 mL) and dried in air. Yield: 265.9 mg, 75.4%. Anal. for FeC_23_H_34_N_9_O_12.5_F_6_S_2_ (870.54 g/mol): calcd. C, 31.73; H, 3.94; N, 14.48; F, 13.09; S, 7.37; Fe, 6.42%. Found C, 31.64; H, 3.76; N, 14.41; F, 12.12; S, 6.37; Fe, 6.59%. IR (KBr, cm*^−^*^1^): υ(C=O)∼1,732(vs), υ(C–O)∼1,211, 1,093(s), υ(C=N)∼1,560(m), υ(C–H out of plane)∼1,028(m), υ(C–H ring torsion)∼632(m), υ_(S–O)_∼1,282, 1,255 (vs, with shoulders).

### 3.3. Physical Measurements

Elemental analyses were performed at University College London (UK) and at S.C.A. CNRS Solaize (France). ^1^H and ^13^C NMR spectra were recorded at 300 MHz and 75 MHz, respectively, on a Bruker AC300 instrument. The residual solvent peak was used as internal reference. Mass spectral data were obtained on Thermo Finnigan LCQ Ion trap spectrometer (APCI mode). HRMS were carried out on a Micromass Q TOF 2 spectrometer in ESI mode, detecting positive mode. Raman spectra with 1,064 nm excitation were recorded between 2,300 to 400 cm^−1^ with a Bruker RFS 100/s FT-Raman spectrometer (*I* = 200 mW) at r.t using a diode-pumped, air-cooled Nd:YAG laser as the excitation source. IR spectra were collected on a Shimadzu FTIR-84005 spectrometer using KBr pellets. Thermogravimetric analyses (TGA) were performed in air (100 mL/min) at the heating rate of 1 °C/min from 293 K to 400 K using a Mettler Toledo TGA/SDTA 851e analyzer. Diffuse reflectance spectra on solids were recorded with a CARY 5E spectrophotometer using polytetrafluoroethylene as a reference. Powder X-ray diffraction patterns were recorded on a Siemens D5000 counter diffractometer working with Cu-Kα radiation and operating at room temperature. The samples were mounted on the support with silicon grease. ^57^Fe Mössbauer spectra were recorded in transmission geometry over the temperature range (78–300 K) with a conventional Mössbauer spectrometer equipped with a Cyclotron Ltd ^57^Co(Rh) radioactive source operating at room temperature. The samples were sealed in aluminum foil and mounted on an Oxford nitrogen bath cryostat. The spectra were fitted to the sum of Lorentzians by a least-squares refinement using Recoil 1.05 Mössbauer Analysis Software [32]. All isomer shifts refer to α-Fe at room temperature. Magnetic susceptibilities were measured in the temperature range 4–390 K using a MPMS-XL (7T) SQUID magnetometer. Data were corrected for magnetization of the sample holder and diamagnetic contributions, which were estimated from the Pascal constants. Differential scanning calorimetry measurements were carried out in a He_(g)_ atmosphere using a Perkin-Elmer DSC Pyris 1 instrument equipped with a cryostat and operating down to 98 K. Aluminum capsules were loaded with 20–50 mg of sample and sealed. The heating and cooling rates were fixed at 10 K min^−1^. Temperatures and enthalpies were calibrated over the temperature range of interest (298–400 K) using the solid-liquid transitions of pure In (99.99%) [33], and the crystal-crystal transitions of pure cyclopentane (≥99%) [34], over the range 78–298 K. Scanning electron microscopy (SEM) was performed using a Gemini Digital Scanning Microscope 982 with 1 kV accelerating voltage with an aluminum sample holder.

## 4. Concluding Remarks

We have presented two novel Fe^II^ 1D ST chain compounds switching in the range 225–250 K. Release of non coordinated water molecules has a paramount effect on the LS state stabilization of **1**. Its air stability and absence of solvent will ease further physical measurements, particularly using hydrostatic pressure [5] so as to shift its hysteresis loop towards the room temperature region.
